# Case Report: Chronic Q fever mimicking malignancy and tuberculosis in a hemodialysis patient: multidisciplinary diagnosis guided by metagenomic next-generation sequencing

**DOI:** 10.3389/fmed.2025.1656891

**Published:** 2025-11-18

**Authors:** Weidong Huang, Hai-ping Lai, Lishi Yu, Lie Jin, Wenhui Lei

**Affiliations:** 1 Department of Nephrology, The Fifth Affiliated Hospital of Wenzhou Medical University, Lishui, Zhejiang, China; 2 Department of Abdominal Surgery, Ganzhou Cancer Hospital, Ganzhou, Jiangxi, China; 3 Department of Rheumatology, The Fifth Affiliated Hospital of Wenzhou Medical University, Lishui, Zhejiang, China

**Keywords:** Q fever, *Coxiella burnetii*, PET-CT, mNGS, hemodialysis, malignancy, tuberculosis

## Abstract

**Background:**

Q fever, caused by *Coxiella burnetii*, is a rare zoonosis whose clinical presentation is highly heterogeneous. Chronic Q fever can present with atypical systemic masses, creating significant diagnostic challenges as it lacks distinctive imaging features, often leading to misdiagnosis.

**Case Presentation:**

We report a case of a 50-year-old woman on maintenance hemodialysis who presented with a one-month history of generalized myalgia and abdominal discomfort. Initial PET-CT imaging revealed multiple hypermetabolic abdominal lesions (SUV∼max∼ 7.1), mimicking metastatic malignancy. Histopathology of abdominal biopsies showed granulomatous inflammation with necrosis but lacked definitive microbiological evidence. Empirical anti-tuberculosis therapy was initiated based on clinical suspicion. Despite initial clinical improvement, the patient experienced recurrence of symptoms and radiological progression after 1 year. Re-evaluation with transesophageal echocardiography suggested the possibility of infective endocarditis. Crucially, metagenomic next-generation sequencing (mNGS) of a repeat biopsy identified *Coxiella burnetii*, confirming chronic Q fever. Targeted doxycycline therapy resulted in sustained clinical and radiological improvement, with lesion resolution confirmed at the 14-month follow-up.

**Conclusion:**

This case underscores the diagnostic difficulty of chronic Q fever due to its non-specific presentation and imaging characteristics. PET-CT may suggest malignancy, but incorporating advanced molecular diagnostics such as mNGS is critical for accurate pathogen identification. Recognizing atypical manifestations and utilizing integrative diagnostic approaches can facilitate timely, targeted therapy, improving clinical outcomes in rare infectious diseases like Q fever.

## Introduction

Q fever is a rare zoonotic infectious disease caused by *Coxiella burnetii*. Its chronic form is characterized by highly heterogeneous clinical presentations and lacks specific imaging features, often leading to interdisciplinary misdiagnoses (1–5). This report describes a case of chronic Q fever presenting primarily with multiple systemic mass lesions. The patient was initially misdiagnosed with metastatic malignancy based on PET-CT, which revealed multiple hypermetabolic lesions in the abdominal cavity (SUVmax 7.1). Subsequent pathological biopsy suggested tuberculosis, but definitive diagnosis was established through metagenomic next-generation sequencing (mNGS), which detected *Coxiella burnetii*-specific sequences from the puncture tissue. Coupled with a review of relevant literature, this case highlights the typical imaging features of chronic Q fever on PET-CT and emphasizes the crucial role of mNGS in establishing the diagnosis. The findings aim to provide valuable reference for radiologists and clinicians in the differential diagnosis and management of Q fever.

## Case presentations

A 50-year-old woman was admitted on March 29, 2023, reporting a one-month history of persistent bilateral waist and back pain, accompanied by abdominal pain, systemic myalgia and constitutional symptoms including chills, subjective low-grade fever (unquantified), fatigue, night sweats, and decreased appetite. Her medical history included a 15-year history of chronic kidney disease (CKD). She had been on peritoneal dialysis for 9 years before switching to maintenance hemodialysis (MHD) 5 years prior due to ultrafiltration failure. She also had hypertension and hypertensive retinopathy, both well-controlled by dialysis; antihypertensive medications had been discontinued following episodes of hypotension. The patient denied recent contact with livestock, wild animals, or tick bites.

On examination, her vital statistics were: height 153 cm, weight 42.5 kg, and body mass index 18.15 kg/m^2^. Physical examination revealed a soft, non-tender abdomen without costovertebral angle tenderness or other systemic abnormalities. Laboratory investigations showed an elevated C-reactive protein (74.51 mg/L) and a mildly elevated procalcitonin (0.15 ng/mL). The erythrocyte sedimentation rate was slightly elevated at 18 mm/h. A chest CT scan was unremarkable. Serological tests ruled out active tuberculosis (T-SPOT.TB negative) and common autoimmune etiologies (autoantibody profile negative). Detailed liver and renal function parameters are presented in Table 1.

**TABLE 1:** Laboratory findings on admission.

The laboratory parameters	Results (reference range)
White blood cells	11.9 × 10^∧^9/L (3.5–9.5)
Hemoglobin	142 g/L (130–175)
Neutrophil percentage	88.1% (40.0–75.0)
Absolute lymphocytes count	0.5 (1.1–3.2)
Platelet	217 × 10^9/L (125–350)
ESR	18 mm/1 h (0–15)
CRP	74.51 mg/L (<8)
Alanine aminotransferase	8 u/L (7–40)
Aspartate aminotransferase	13 u/L (13–25)
Total bilirubin	4.2 umol/L (<23.0)
Albumin	35.7 g/L (40–55)
Creatinine	384 umol/L (57–111)
Procalcitonin	2.31 (< 0.05)
ANA	Negative
ANCA	Negative
Anti GBM antibody	Negative
T-SPOT.TB	Negative

**FIGURE 1:**
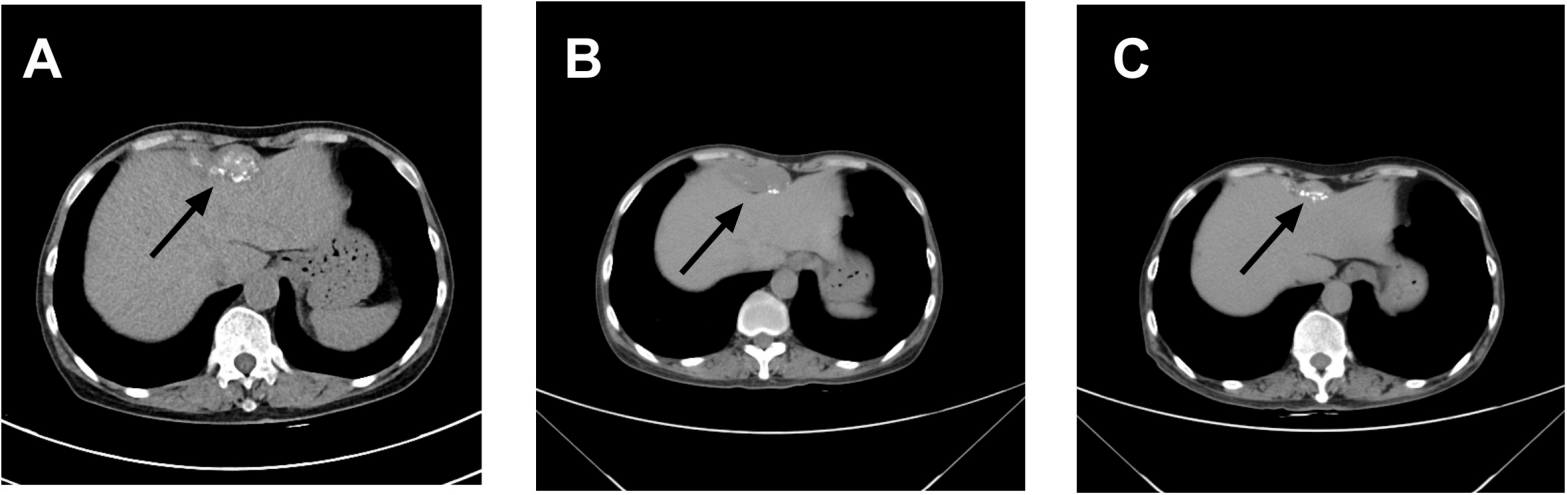
Changes in abdominal mass observed on computed tomography (CT): **(A)** On April 8, 2023, contrast-enhanced abdominal CT revealed an irregular soft tissue density lesion anterior to the liver, measuring 5.2 × 2.3 × 6.2 cm. A small lymph node (∼1.5 cm) was noted at the level of the umbilicus in the left abdominal cavity, with a consideration of malignant tumor. **(B)** On March 15, 2024, contrast-enhanced abdominal CT demonstrated a calcified mass anterior to the liver (5.1 × 2.0 × 3.6 cm) and a small lymph node (∼0.8 × 0.8 cm) at the same level within the left abdominal cavity, with a differential diagnosis of abdominal tuberculosis. **(C)** On October 30, 2024, contrast-enhanced abdominal CT showed a smaller anterior hepatic mass with calcification (3.9 × 1.3 cm). The lymph node at the umbilical level in the left abdominal cavity remained unchanged (∼0.8 × 0.8 cm). Arrows indicate the lesion locations.

**FIGURE 2:**
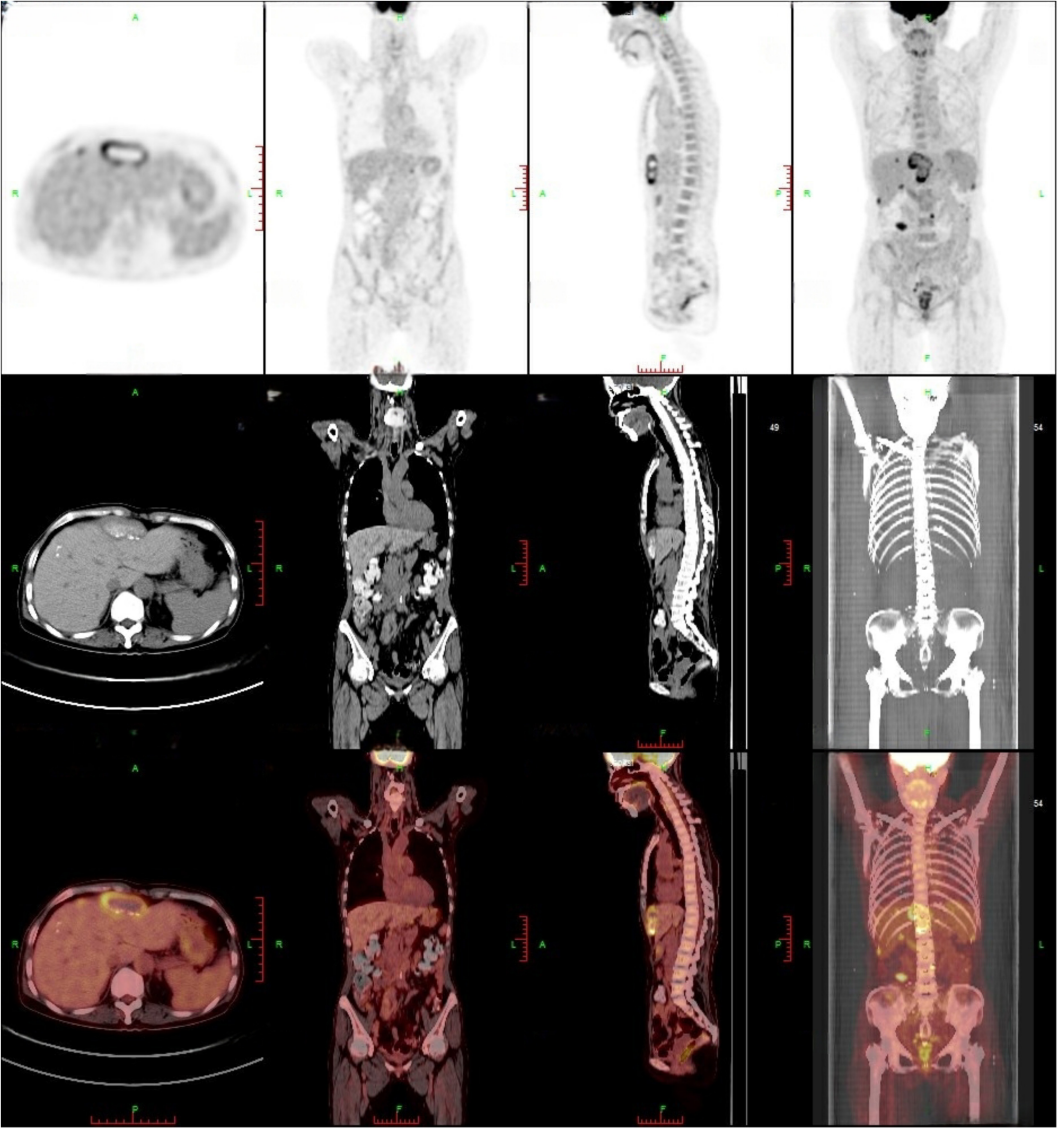
PET-CT findings: multiple peritoneal nodules and masses (approximately 5.5 × 2.2 cm) exhibited increased FDG accumulation, with a maximum SUV of 7.1, suggesting peritoneal metastasis. A high-uptake lesion beneath the right abdominal wall was recommended for percutaneous biopsy.

Contrast-enhanced abdominal CT (Figure 1) revealed an irregular soft-tissue density lesion (approximately 5.2 × 2.3 × 6.2 cm) anterior to the liver, accompanied by a 1.5 cm nodule in the left peritoneal cavity, raising suspicion of malignancy. Subsequent PET-CT (Figure 2) confirmed multiple FDG-avid peritoneal nodules and masses, with a maximum standardized uptake value (SUV∼max∼) of 7.1, further supporting the suspicion of metastatic disease. Blood and urine cultures showed no growth.

**FIGURE 3:**
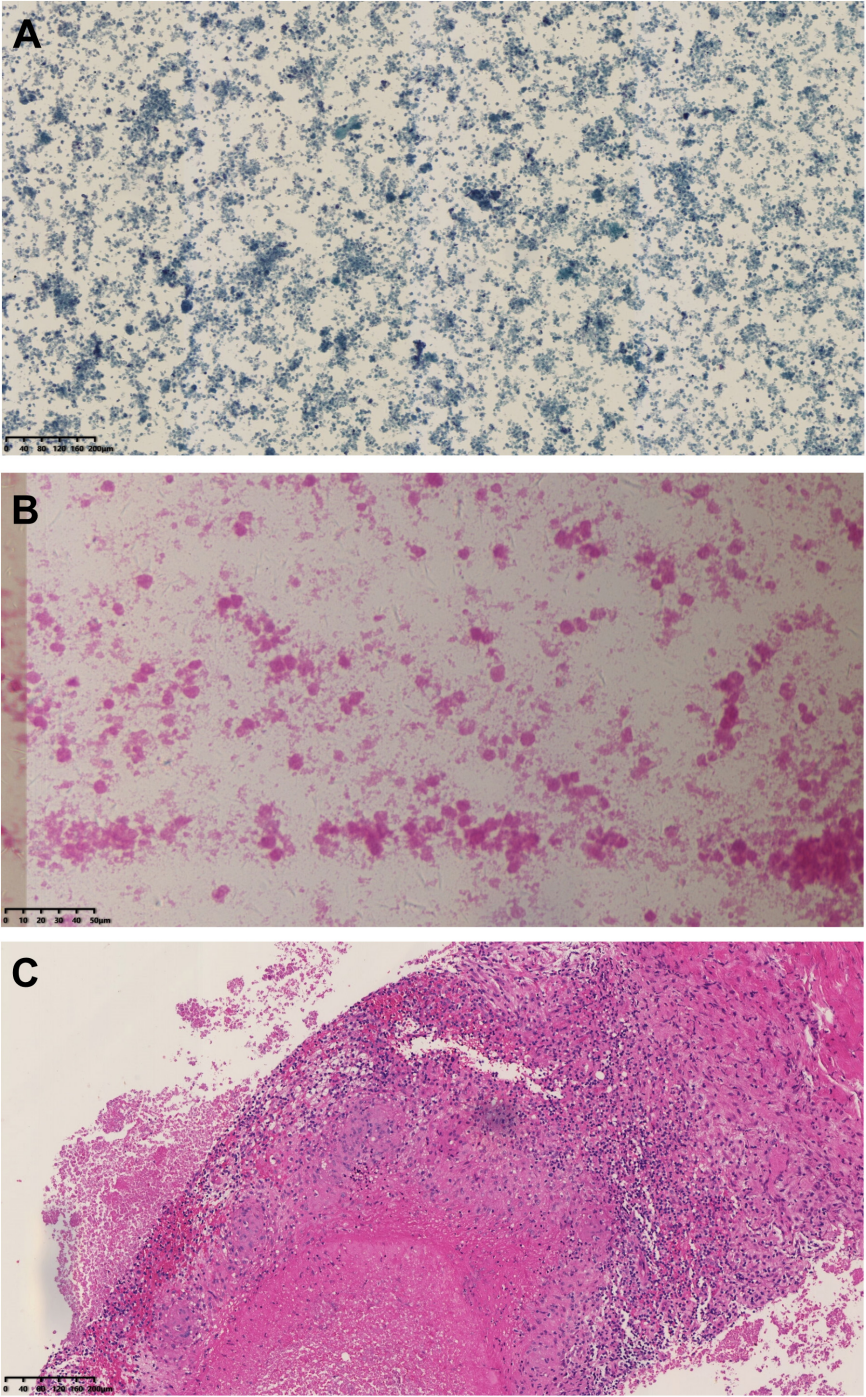
Histopathological results of abdominal mass biopsy: **(A)** Papanicolaou (PAP) stain at 100 × magnification and **(B)** hematoxylin and eosin (H&E) stain at 400 × magnification of the biopsy obtained on April 13, 2023, revealing basophilic amorphous deposits. **(C)** HE stain at 100 × magnification from the biopsy performed on March 20, 2024, demonstrated granulomatous inflammation with necrosis.

Initial empirical antibiotic therapy with ceftriaxone (2 g daily) and moxifloxacin (0.4 g daily) was administered. However, the patient’s body temperature remained elevated (37.5–38°C). An ultrasound-guided biopsy of the peritoneal lesion was performed on April 13, 2023, during which approximately 8 mL of caseous pus was aspirated. Histopathological examination demonstrated granulomatous inflammation with multifocal necrosis (Figure 3). Microbiological studies, including acid-fast staining, GeneXpert MTB/RIF assay, and tuberculin skin test, were all negative.

Given the patient’s immunocompromised status due to long-term dialysis and the histopathological findings of caseous necrosis, infectious etiologies—particularly tuberculosis—were strongly suspected. Following multidisciplinary consultation, a diagnosis of tuberculous peritonitis was considered, and an empirical anti-tuberculosis regimen was initiated with isoniazid (0.3 g daily), rifampin (0.45 g daily), pyrazinamide (1.5 g thrice weekly), and ethambutol (0.75 g thrice weekly). This regimen was poorly tolerated, with severe gastrointestinal adverse effects (nausea, vomiting, abdominal pain) emerging within 1 week, attributed to pyrazinamide and ethambutol. These two drugs were discontinued and replaced with moxifloxacin (0.4 g daily), resulting in a revised regimen of isoniazid, rifampin, and moxifloxacin. The patient was discharged on April 21, 2023, and continued her regular hemodialysis. At the one-month follow-up, she reported symptomatic improvement, with decreased myalgia and fewer febrile episodes. Inflammatory markers had also improved (CRP: 38 mg/L; ESR: 12 mm/h), suggesting an initial treatment response.

**FIGURE 4:**
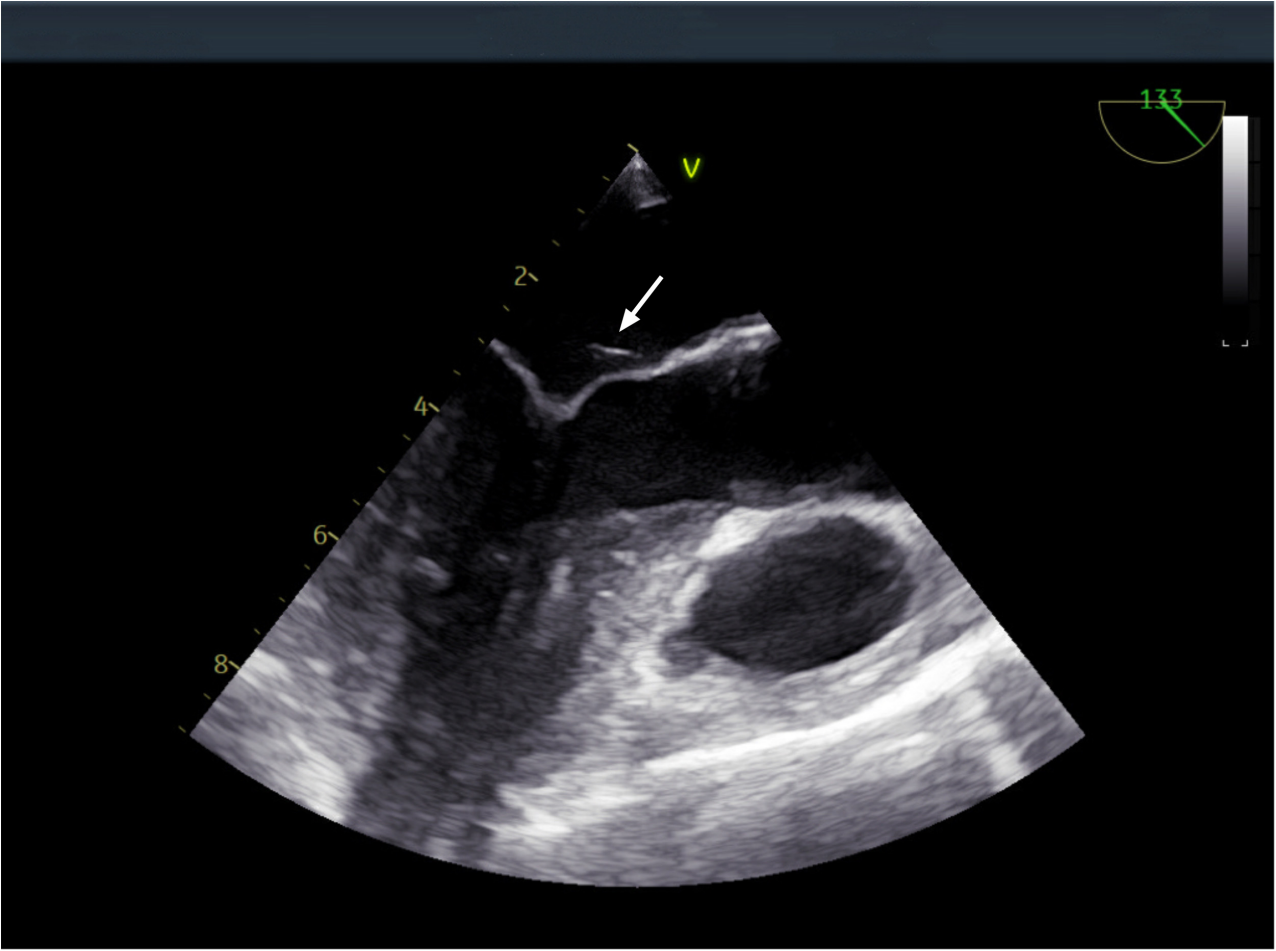
Transesophageal echocardiography: a vegetation on the mitral valve was detected. The arrow indicates the location of the mitral valve vegetation.

However, after completing 1 year of anti-tuberculosis therapy, the patient was re-admitted on March 15, 2024, due to recurrent low-grade fever. Repeat abdominal CT (Figure 1) showed progression of the hepatic lesion (now 5.1 × 2.0 × 3.6 cm) and persistent peritoneal thickening. Transesophageal echocardiography (TEE) revealed vegetations on the mitral valve (Figure 4), compatible with infective endocarditis.

A repeat ultrasound-guided biopsy of the abdominal lesion was performed on March 20, 2024. Histology again showed granulomatous inflammation with focal necrosis (Figure 3), and acid-fast staining remained negative. Crucially, metagenomic next-generation sequencing (mNGS) analysis of the biopsy tissue detected *Coxiella burnetii*-specific sequences, with 66 unique reads and a relative abundance of 66.01%. Based on these findings, the patient’s dialysis history, and immunocompromised status, a definitive diagnosis of chronic Q fever with infective endocarditis was established. Anti-tuberculosis medications were immediately discontinued, and targeted therapy with doxycycline (0.1 g twice daily) was initiated. The patient was discharged on March 27, 2024, on doxycycline monotherapy.

As of June 2025 (14 months post-treatment initiation), the patient remained symptom-free, with no recurrent fever, myalgia, or fatigue. A follow-up abdominal CT on October 30, 2024, showed significant reduction of the Intra-abdominal lesion to approximately 3.9 × 1.8 × 1.2 cm and marked resolution of peritoneal thickening, confirming effective treatment (Figure 1). She continues regular outpatient follow-up, maintaining good mental health and nutritional status, with no signs of disease recurrence.

## Discussion

*Coxiella burnetii*, the pathogen responsible for Q fever, was first isolated in Australia in 1937 (6). As an obligate intracellular Gram-negative bacterium, *Coxiella burnetii* exhibits extensive host range-including cattle, sheep, domestic pets, and wild animals, surviving for months in dust and other fomites. These features have contributed to its status as one of the most widely distributed zoonotic pathogens worldwide (7, 8). Notably, China, being a major livestock-producing country, has observed an increasing incidence of chronic Q fever, particularly among immunocompromised populations. The present case of a patient undergoing maintenance hemodialysis (MHD) exemplifies this high-risk group.

The clinical manifestations of Q fever are highly heterogeneous. Approximately 60% of infected individuals remain asymptomatic, with diagnosis often relying solely on serological screening (9, 10). Acute Q fever typically presents as a triad of fever, headache, and myalgia. Conversely, chronic infection predominantly manifests as endocarditis (accounting for 60–70% of chronic cases), vascular infections, or osteoarticular conditions (11). This case highlights several diagnostic challenges: (1) Atypical clinical presentation: The patient primarily exhibited systemic myalgia and low-grade fever, lacking specific respiratory or gastrointestinal symptoms. (2) Absence of a clear epidemiological history: The patient denied direct contact with animals, which could lead to underestimation of zoonotic transmission risk. (3) Limitations of traditional diagnostic methods: Serological testing is hindered by the diagnostic window period, during which IgM and IgG antibodies may be undetectable for 2–4 weeks post-infection (12). Culture-based diagnosis requires biosafety level III laboratories with specialized media, which are limited in most tertiary hospitals in China. Furthermore, culture has a low positive rate and is time-consuming, restricting its practical application in clinical settings (13, 14). Serological testing for Q fever (IgM and IgG antibodies) was not performed due to lack of assay availability at our institution.

In this case, the initial whole-abdomen enhanced CT scan revealed a hepatic anterior mass and multiple intra-abdominal nodules. Subsequent PET-CT demonstrated multiple FDG-avid lesions in the peritoneum, with a maximum standardized uptake value (SUVmax) of 7.1. These imaging features closely mimicked malignant neoplasms, particularly peritoneal metastases. Literature suggests that 18F-FDG PET/CT can detect focal hypermetabolism on cardiac valves or vascular walls, facilitating diagnosis of infectious endocarditis and vascular infections in chronic Q fever (15). This modality improves diagnostic accuracy when incorporated into the Duke criteria and helps localize sites of chronic Q fever infection (16, 17). In the present case, however, PET-CT demonstrated increased metabolic activity initially suggestive of multifocal metastatic malignancy rather than Q fever. Subsequent percutaneous biopsy revealed only basophilic amorphous material and granulomatous inflammation with negative acid-fast staining. These non-specific findings provided no definitive microbiological evidence and led to the initial misdiagnosis of tuberculous peritonitis.

Metagenomic next-generation sequencing (mNGS) played a pivotal role in the definitive diagnosis of this case. By performing unbiased sequencing directly on the biopsy tissue, mNGS detected *Coxiella burnetii*-specific sequences with a relative abundance of 66.01%, overcoming the limitations of traditional diagnostic methods that heavily rely on presumptive criteria (14, 18). This technique is particularly advantageous in the following scenarios: (1) opportunistic infection screening in immunocompromised populations; (2) cases with suspected infection where conventional pathogen detection yields negative results; and (3) critically ill patients who require rapid identification of the infectious source to guide targeted therapy.

Compared to traditional methods, mNGS circumvents the need for culture by using high-throughput sequencing to rapidly and objectively identify pathogens within clinical samples. This is particularly advantageous for diagnosing complex cases. Over recent years, mNGS has been increasingly applied to detect rare or fastidious pathogens. For instance, during the COVID-19 pandemic, it effectively identified co-infections with fungi, viruses, or bacteria (19). Wilson et al. reported a case of *Babesia* meningoencephalitis diagnosed via mNGS (20), while Mai et al. detected Japanese encephalitis virus in a 16-year-old boys urine (21). These studies demonstrate that mNGS significantly enhances pathogen detection, especially for rare microorganisms. In this case, imaging suggested malignancy, but repeated pathogen testing was negative, and exclusion of tumors and autoimmune diseases prompted consideration of rare infections. Ultimately, mNGS identified *Coxiella burnetii*, guiding targeted therapy and resulting in clinical improvement.

During the patients second hospitalization, TEE demonstrated a mitral valve vegetation. In combination with metagenomic next-generation sequencing (mNGS) results, the diagnosis was suspected to be Q fever endocarditis. Q fever endocarditis is often associated with pre-existing valvular heart disease, immunosuppression, or pregnancy (22–24). Currently, doxycycline is the first-line treatment for Q fever (6),but in cases complicated by infective endocarditis, adjunctive hydroxychloroquine is recommended (25). Given this patient’s hypertensive retinopathy, doxycycline monotherapy was chosen, and the patient has been monitored for 14 months without recurrence.

This case report has several limitations. First, as a single-case study, its findings lack generalizability. The diagnostic process was constrained by the limited availability of specific tests at our institution. For instance, serological testing for *Coxiella burnetii* antibodies-the gold standard for diagnosing chronic Q fever-was not performed. Consequently, the absence of this critical data meant we could not provide serological confirmation or phase-specific antibody titers to fully satisfy the modified Duke criteria for infective endocarditis. Therefore, the diagnosis relied heavily on the positive mNGS result from the local lesion, which, while highly suggestive, cannot definitively exclude the possibility of a coincidental presence of the pathogen. Furthermore, the route of transmission remains unclear, as the patient lacked a key epidemiological risk factor: documented animal exposure.

## Conclusion

In conclusion, chronic Q fever caused by *Coxiella burnetii* presents considerable diagnostic challenges in clinical practice due to its non-specific clinical presentation and routine laboratory findings. This case underscores that imaging features, including contrast-enhanced CT and PET, may demonstrate multifocal hypermetabolic lesions that mimic metastatic malignancy, potentially leading to misdiagnosis. In such scenarios where imaging cannot reliably distinguish between infectious and malignant processes, close collaboration between radiologists and clinicians—supplemented by careful epidemiological assessment—is crucial. Moreover, this case demonstrates that metagenomic next-generation sequencing (mNGS) can serve as a valuable diagnostic tool for rapidly identifying fastidious pathogens such as *Coxiella burnetii*, thereby facilitating earlier targeted antimicrobial intervention. Further studies are warranted to validate the diagnostic utility of mNGS in suspected cases of chronic Q fever and to establish its role in the diagnostic workflow when conventional methods yield inconclusive results.

## Data Availability

The original contributions presented in this study are included in this article/supplementary material, further inquiries can be directed to the corresponding author.
